# Profiling adaptive immunity: A quantitative framework for immune repertoire dynamics and clinical diagnostics

**DOI:** 10.1016/j.fmre.2025.05.011

**Published:** 2025-06-03

**Authors:** Yexing Chen, Haiwen Ni, Yongjie Li, Jin Ma, Chen Huang, Sixian Yang, Xiangfei Xie, Haitao Lv, Min Li, Peng Cao

**Affiliations:** a State Key Laboratory of Technologies for Chinese Medicine Pharmaceutical Process Control and Intelligent Manufacture, Nanjing University of Chinese Medicine, Nanjing 210000, China; b Jiangsu Provincial Medical Innovation Center, Affiliated Hospital of Integrated Traditional Chinese and Western Medicine, Nanjing University of Chinese Medicine, Nanjing 210000, China; c Department of Hematology, Affiliated Hospital of Nanjing University of Chinese Medicine, The First Clinical College of Nanjing University of Chinese Medicine, Nanjing 210000, China; d Children’s Hospital of Soochow University, Suzhou 215000, China; e Key Laboratory of Quantum Information, University of Science and Technology of China, Hefei 230000, China; f Department of Computer Science, College of Engineering and Applied Science, University of Cincinnati, Cincinnati 45221, USA; g Department of Oncology, Nanjing Hospital of Chinese Medicine Affiliated to Nanjing University of Chinese Medicine, Nanjing 210000, China

**Keywords:** Deductive reasoning, Immune repertoire analysis, Repertoire shift quantification, Early disease screening, Systemic immunity inference, Kawasaki disease, Colorectal cancer

## Abstract

While immune repertoire (IR) dynamics encode critical disease signatures, existing models fail to capture the non-equilibrium processes that govern clonal selection and somatic hypermutation (SHM). We develop a biophysical framework that mathematically reconstructs IR evolution through energy landscape optimization, where 1) clonal emergence probabilities map to metastable states, 2) repertoire transitions obey non-equilibrium dynamics, and 3) inter-IR distances quantify the distribution transformation costs via optimal transport theory. This model enables macroscopic immune state detection from 10k cells by resolving critical fluctuations in sparse sampling regimes. Experimental validation across murine/human cohorts demonstrates precise unsupervised stratification of immune stages and disease states without prior clinical annotations. By bridging stochastic SHM kinetics with deterministic repertoire shifts, our approach establishes quantitative metrics for tracking immunological trajectories and pathological progression. This paradigm shift from static pattern recognition to dynamic process decoding empowers sensitive immunomonitoring and personalized therapeutic design through ultra-low-input clinical sampling.

## Highlights

• The article derives first-principle relationships from foundational biological assumptions, grounding the approach in deductive reasoning rather than data mining.
• BCR repertoire diversity is modeled through generative complexity, and repertoire shift is defined as the minimum energy cost to transition one distribution to another.
• This study provides theoretical and mathematical foundation for the observed phenomenon of B and T cells such as scale-free distribution.
• This study proposes a generalizable disease differentiation framework that achieves precise discrimination between Kawasaki disease (KD), colorectal cancer(CRC), and healthy cohorts upon first exposure to untrained data.
• This study establishes sample size-independent immune repertoire profiling and enables clinically precise discrimination of individuals across age strata, infection histories, KD, and CRC using minimal sample volumes.

## Graphical abstract

**Figure:**
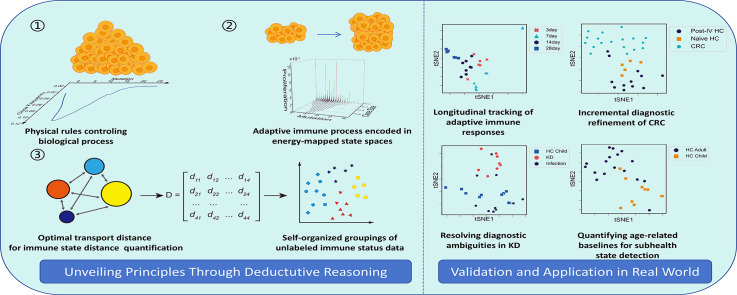

## 1 Introduction

In the history of physical and chemical sciences, the research trajectory has typically followed a dual-phase paradigm. The initial phase employs bottom-up data-mining approaches that derive empirical patterns from phenomenological observations, exemplified by discoveries such as Kepler’s Laws of Planetary Motion, Boyle’s Law, and affinity tables in early chemistry. Once foundational knowledge becomes established, research transitions to top-down logico-deductive methodologies rooted in axiomatic principles, as demonstrated by Newtonian mechanics, Maxwell-Boltzmann distribution for ideal gases, and thermodynamic formalisms describing chemical kinetics. This theoretical paradigm operates through predictive verification methodology by deriving formulae prior to empirical validation, which eliminates reliance on statistical induction. Empirical laws provide critical data anchors for theoretical construction, while theoretical models offer predictive frameworks for novel phenomenon discovery. The iterative progression between these two paradigms constitutes the fundamental dynamic of disciplinary advancement. Current immunological research, particularly in T and B-cell maturation studies, stands at this critical juncture of transitioning from phenomenological accumulation to theoretical systematization, necessitating urgent development of mathematical frameworks tailored to the complexity of immune systems.

The adaptive immune system mounts a specific response to antigens including pathogens such as bacteria and viruses, as well as in autoimmune diseases and cancer. Adaptive immunity is triggered when a threshold strength of signals is reached [1]; this process may occur before the manifestation of any clinical symptoms. Adaptive immunity exhibits distinct response characteristics under the stimulation of different antigens [2], so by detecting the activation and characteristic of adaptive immunity, early disease diagnosis and etiological screening may be achieved. Antigen recognition in adaptive immune system is achieved through B cells and T cells by their respective receptors, the B cell receptor (BCR) and T cell receptor (TCR). These receptors exhibit a high degree of diversity to ensure compatibility with the diverse range of antigens [3], [4]. Immune repertoire refers to the concept of the functional assemblies of BCR and TCR receptor, which shifts due to the selection of high-affinity cells during the adaptive immune process.

Central challenges in quantitative repertoire analysis include the dynamic description of repertoire generation [5], summarizing the diversity of the entire repertoire [6], the computation of the repertoire shift [7], as well as the correlation between disease and repertoire behavior [8]. Several approaches have been developed to depict the repertoire by modeling sequencing results, such as the diversity measurements [6], similarity aggregating methods [9], and artificial intelligence [10]. Current analytical strategies often limit the scope to either tracking diversity origins during lymphocytic differentiation or capturing static repertoire snapshots. While these approaches remain confined to phenomenological pattern mining through correlative metrics (e.g., Shannon entropy-health status associations), the causal drivers of repertoire evolution remains not fully studied. Quantitative repertoire analysis still has three intrinsic limitations: (i) scale-dependent artifacts necessitating cohort-specific normalization (Extended Data Fig. 1), (ii) directional blindness to clonal trajectory progression, and (iii) magnitude ambiguity in quantifying immunodynamic potentials. This methodology traps researchers in post-hoc correlative inference, systematically obscuring the non-equilibrium dynamics principles that orchestrate repertoire state transitions. We posit that transcending this descriptive stalemate requires process-explicit modeling, capable of resolving real-time clonal selection energetics, quantifying immune adaptability through optimal transport theory, and forecasting pathogenic progression via phase-space trajectory analysis; then finally a causally grounded framework for mechanistic immunodynamics is established.

**Fig. 1:**
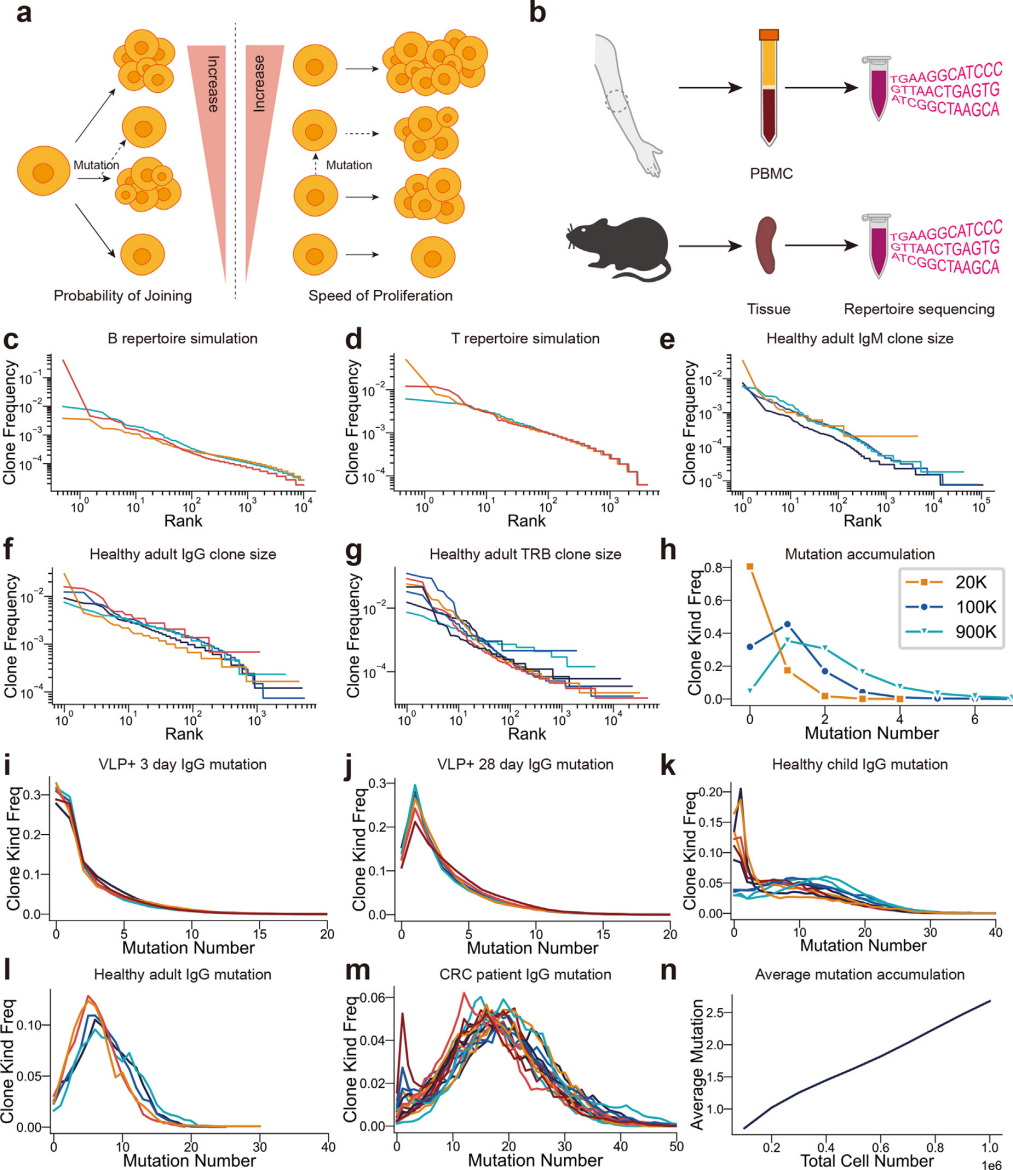
**The preferential attachment model simulate the rank-frequency and mutation distributions of repertoire.** Each line represents an individual or a simulation instance. (a) Clones with high proliferation speed equals to have high probability to attract new cells to attach to them. New clones are generated when mutation occurs. (b) Samples are collected from human peripheral blood and mouse spleen, and bulk HTS sequencing are used to get repertoire data directly from total RNA without FACS sorting or single cell techniques. (c, d) BCR&TCR repertoires generated from the model show the scale free characteristics. (e, f, g) The scale free characteristics of IgM, IgG, and TRB repertoire data from healthy adults. (h) The simulated distribution of mutation-clone shifts with different repertoire size, processes involving proliferation exhibit higher average mutation loads. (i, j) C57BL/6 mice are immunized with VLP. The mutation-clone curve in early (3 days) and late (28 days) stages shows similar distribution with the simulated repertoire. (k, l, m) The mutation-clone curve in clinical samples. The unexperienced(healthy child), experienced(healthy adult) and well activated(cancer patients) immune system also show the similar unimodal and shift pattern as in the simulated repertoire. (n) In the simulated repertoire, mutation accumulates linearly with the proliferation process.

Capitalizing on contemporary IR datasets of unprecedented scope, our analytical focus now transitions from isolated feature characterization to decoding self-organizing cellular population dynamics. This strategic realignment seeks to elucidate conserved organizational logic and universal biomathematical principles governing IR-mediated recognition across systemic hierarchies. We implement a multi-scale computational pipeline: (1) encoding High Throughput Sequencing (HTS) derived repertoires into energy-mapped state spaces where immunological accessibility is quantified via metadynamics simulations of transition-state energetics; (2) formulating repertoire plasticity as non-equilibrium dynamics processes, with state transitions characterized by optimal transport theory; (3) validating first-principle predictions through longitudinal repertoire tracking across antigen challenge cohorts. This approach not only resolves prior observational paradoxes but establishes causal linkages between microscopic receptor dynamics and macroscopic immune responses. Crucially, validation transcends empirical curve-fitting—stress-testing the model’s explanatory power through novel antigen exposure experiments and cross-species conservation analyses. The emergent scaling laws and universal critical exponents we derive directly operationalize the biophysical principles hypothesized in the preceding theoretical framework, demonstrating quantitative continuity from molecular recognition energetics to population-level immune adaptability. This strict theory-experiment dialog positions our model as a candidate law-like formulation in immune system physics, bridging the historical gap between immunological complexity characterization and predictive theoretical synthesis.

## 2 Methods

### 2.1 Building repertoire quantification model

#### 2.1.1 Quantitative visualization of the repertoire distribution

Previous experimental evidence has documented divergent IgG antibody gene usage frequencies and IgG-RBD/NP titers in SARS-CoV-2 infected monozygotic twins [11], indicating substantial non-heritable determinants in emergent BCR and TCRβ clonal dominance. This phenotypic divergence has been attributed to stochastic competition during lymphocyte activation, where antigen presentation timing imposes first mover advantage in activation dynamics on naive cells within the competent repertoire. Consequently, structural epitope-focused analyses exhibit limited utility in identifying conserved immune signatures across individuals.

Notably, while antigen-specific responses generate distinct dominant BCR or TCR sequences, they universally employ conserved cellular expansion and maturation mechanisms. This paradox motivated developing a quantitative framework characterizing immune response convergence through process-oriented metrics: (1) receptor diversity (BCR/TCR clonotype counts), (2) clonal expansion hierarchy (UMI-distinct sequence per clone), and (3) affinity maturation progression (BCR mutation loads relative to germline sequences). These parameters, directly derivable from bulk sequencing data (clonotype counts, UMI-collapsed sequences, and SHM-derived mutation counts), have been incorporated into our computational framework to model conserved lymphocytic selection dynamics.

We established a computational 3D coordinate system with axes representing somatic hypermutation number (X), clonal diversity (Y), and proliferative expansion (Z) (Extended Data Fig. 2). Clones were defined by identical BCR heavy chain or TCRβ chain nucleotide sequences for B and T cells, respectively. Normalizing Y and Z axes to unit scales (0-1) enables precise spatial mapping, where each cell occupies a unique coordinate reflecting its mutational history and clonal abundance. This framework systematically integrates established repertoire principles: SHM-driven affinity maturation manifests as rightward X-axis shifts [12], mutation hotspots emerge as X-axis density clusters beyond Poisson expectations [13], and epitope spreading is visualized through Z-axis dominance of low-X clones [14]. The geometric arrangement of points within this space encodes disease-specific immunological trajectories, motivating its adoption as our foundational representational schema.

**Fig. 2:**
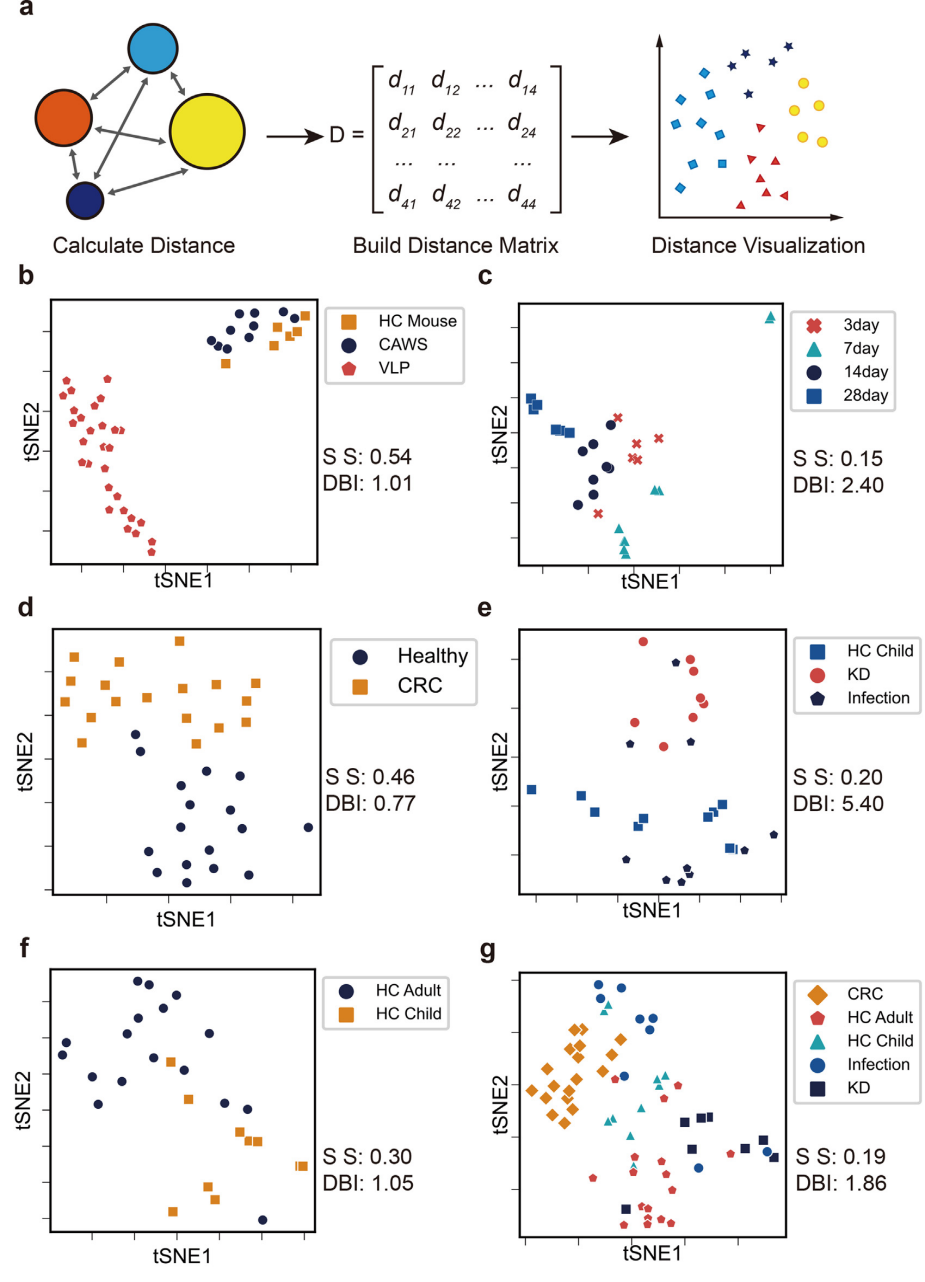
**Repertoire shift calculation and sample clustering based on repertoire distance.** Each point represents an individual. (a) Sample distances are calculated as minimum efforts required to transform one repertoire distribution to another between any two samples. Distance matrix are calculated and then blindly clustered and visualized by t-SNE. (b) Distinct clusters are shown between C57BL/6 mice samples immunized with CAWS and VLP. (c) The clustering method is capable of distinguishing different immune stages in mouse VLP immunization progress. (d) The primary diagnosed CRC shows a unique cluster result. indicating a potential detection method for cancer early diagnosis. (e) The different clustering of KD and infection may help solve the dilemma of misdiagnosis and stop disease progression in early stages. (f) The differences in healthy child and healthy adult clustering shows that the method also depicts dynamic changes in immune system development without antigen stimulation. (g) Clustering of clinical samples with all healthy conditions included in the study, each condition has unique immune pattern and clusters separately.

While pathological states generate characteristic 3D repertoire signatures that yield diverse bioindicators, such disease-specific indices remain inherently empirical and lack generalizable quantification. We instead pursue universal metrics by analyzing inter-repertoire distributional divergence rather than cataloguing phenotypic associations. Central to this approach is resolving two mechanistic questions: (1) What energetic cost governs immune system navigation to specific 3D coordinates? (2) How can we mathematically articulate the transformation effort between repertoire configurations? This paradigm shift from descriptive pattern recognition to dynamic process modeling enables principled quantification of immune adaptation dynamics.

#### 2.1.2 Calculating the difficulty to generate a repertoire

To quantify clonal selection energetics, we implemented the preferential attachment network growth mechanism first formalized by Barabási and Albert [15] to simulate the immune repertoire evolution through biased clonal expansion, wherein newly introduced nodes connect preferentially to existing nodes with higher connectivity. This process operates through two fundamental principles: network growth (gradual node addition) and preferential linkage (connection probability proportional to existing nodes’ degree). The resultant networks exhibit scale-free architecture characterized by power-law degree distributions, a pattern ubiquitously observed in social and economic systems.

In the context of B/T cell activation dynamics, this framework finds biological correspondence: Naive lymphocytes with diverse BCR/TCR repertoires constitute the initial network nodes, while clonal expansion events mirror the addition of new links preferentially directed toward immunodominant clones (Fig. 1a). This framework translates biological processes including VDJ recombination [16], SHM-driven affinity optimization, and antigen-mediated selection into six core parameters (four for TCR) governing system dynamics (Extended Data Fig. 3):

1. Antigenic stimulation probability distribution.
2. Initial responding lymphocyte population size.
3. SHM efficiency (non-lethal mutation rate) [17].
4. Stimulation signal modulation amplitude per mutation.
5. Proliferation kinetics as sigmoidal functions of stimulation intensity.
6. Clonal diversification through entire process.

**Fig. 3:**
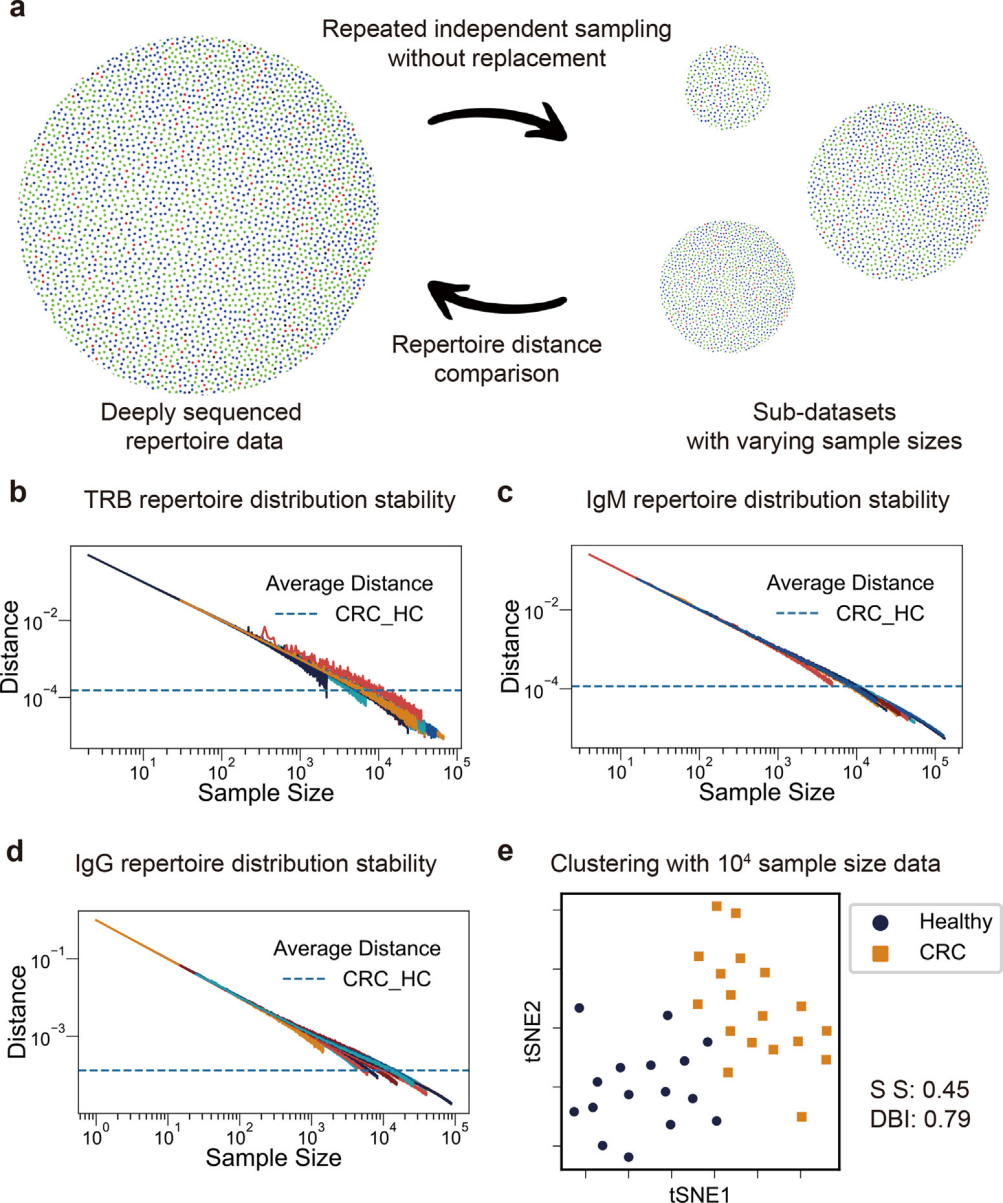
**Structural characteristics of repertoire.** (a) A schematic diagram of the workflow for sample resampling and comparative analysis. Each point corresponds to an individual lymphocyte, with chromatic encoding reflecting its clonal identity. (b, c, d) Each line or point represents an individual. The distances between the overall sequenced dataset and small data subsets resampled from it are calculated. Distances decrease as the sample size increase. Dashed lines are the average distance between healthy adults and cancer patients. The discrepancy between the data subset and the overall population decreases at a power-law rate with the increase of the sample size in IgM, IgG and TRB. (e) Data clustering of CRC and HC samples. Data size are limited to 1$*10^{4}$. The clustering result are consistent with that of full sample size.

In modeling B/T cell receptor diversification mechanisms, the V(D)J recombination process generates a vast diversity of BCR and TCR clonotypes, with only a minimal subset exhibiting antigen-specific responsiveness. This fundamental immunobiological constraint creates maximum-diversity receptor distributions that inherently follow Gaussian characteristics. Mechanistically, the somatic hypermutation process is mathematically conceptualized as Brownian motion along a one-dimensional binding affinity axis. BCR variants undergo stochastic migration governed by Gaussian-distributed step sizes, with evolutionary selection preserving sequences demonstrating positive affinity drift (Δx>0). Both the V(D)J diversification phase and affinity maturation process were consequently modeled using Gaussian probability frameworks, reflecting the intrinsic stochasticity of lymphocyte receptor optimization dynamics.

The adaptive immune response is modeled as a discrete time stochastic process:

1. Initialization: Generate baseline repertoire from parameterized distributions
2. Iterative propagation for each cell:
  (a) Compute SHM-induced stimulation signal perturbation
  (b) Determine proliferation probability via selection thresholds
  (c) Generate progeny with inherited/mutated receptor profiles
3. State monitoring: Track system clonal dominance and energy landscape reconfiguration at each timestep

This architecture captures the dynamics of repertoire evolution, where preferential attachment drives the system toward antibody maturation.

To establish clinical-translational validity through real-world immunological heterogeneity, we integrated preclinical Candida Albicans Water Soluble fraction (CAWS) or Virus-like particle (VLP) [18] challenged murine spleen repertoires with clinical peripheral blood cohorts spanning healthy controls (non-curated, HC, n=17), active infections (n=10), Kawasaki disease (KD, n=8), and colorectal cancer (CRC, n=18) (Fig. 1b), while intentionally retaining natural population complexities—including recent exposures or vaccination in controls and comorbidities in cancer groups. BCR heavy chains (IgA/IgM/IgG) and TCRβ sequences underwent UMI-deduplicated deep sequencing, generating repertoire profiles across acute/chronic immune challenges. This uncurated sampling strategy preserved confounding biological noise to rigorously stress-test model generalizability against realistic clinical variability.

The rank-frequency curve of the simulated repertoire exhibits a fat-tail power law distribution [19] (Fig. 1, c-d), which is consistent with the B-A preferential model that produces a scale-free network. This power law distribution is also observed in real data (Fig. 1, e-g) and previous research results [20]. The scale-free pattern can be considered as the dimension reduction of the 3D structure on Y-Z plane after sorting (Extended Data Fig. 4). The distribution of clones with varying mutation numbers, which is also the projection of the 3D structure on the X-Y plane, distributes an unimodal curve (Extended Data Fig. 4), and both the average and median number of mutations per clone increase with the total number of cells (Fig. 1h). These trends are observed in different immune stages (Fig. 1i-j) and health conditions (Fig. 1k-m, Extended Data Fig. 5a) with significant consistency. The correlation between total cell number $N_{\text{total}}$ and average mutation number per cell $N_{\text{Mut}}$ in the model is almost linear (Fig. 1n).

The consistency of results strongly implies a functional relationship between number of mutations and clone kind, suggesting that the analysis of the immune repertoire should not only be sequence-based but may also need consideration at the clonal aspects, where the clone size could be considered as a parameter of the clone in calculating the mutation rate, proportion, and biological function weight.

Drawing upon mechanical analogies within a phenomenological modeling framework, we propose that the immune stimulation which cause a continual proliferative expansion of immune cells ($N_{\text{total}}$) under antigenic challenge can be conceptualized as an equivalent mechanical ”force”, while the somatic hypermutation load ($N_{\text{Mut}}$) represents the system’s effective displacement in BCR configuration space. This formulation yields a quadratic relationship between mutation burden and required energy input ($E_{\text{SHM}}\propto N_{\text{Mut}}^{2}$), mirroring Hookean elasticity where stored potential energy scales with the square of displacement ($U=0.5kx^{2}$).

The baseline term 0.1 in Eq. 1 serves dual purposes: (1) As a computational regularization parameter, it circumvents undefined energy states for unmutated cells (e.g., TCR-bearing lymphocytes and IgM+ B cells in primary responses) while maintaining dimensional consistency; (2) Phenomenologically, it represents the ground state energy expenditure associated with fundamental cellular homeostasis, distinct from mutation-dependent energy investment. This constant was empirically initialized at 0.1 pending future parameter optimization through experimental calibration.

(1) $$E_{\text{SHM}}=\int N_{\text{Mut}}*N_{\text{total}}dN_{\text{total}}\propto N_{\text{Mut}}^{2}∼N_{\text{Mut}}^{2}+0.1$$

The X axis in the 3D space defines the weight of the clone; points with identical e x-coordinate possess identical weight levels. The spatial distributions exhibits the direction and extent of how immune system react to different immunogenic stimuli, and can serve as an indicator for disease diagnosis.

#### 2.1.3 Repertoire shift quantification

By defining clonal weights through our energy-based metric ($E_{\text{SHM}}∼N_{\text{Mut}}^{2}+0.1$; Eq. 1), we quantify repertoire shifts as the optimal transformation energy between multidimensional clonal distributions. The rationale stems from evolutionary biomechanics: immune cell activation pathways under prolonged selection pressure are hypothesized to converge toward energy-optimal configurations, as resource-efficient activation (e.g., TCR signaling cascades with minimized ATP consumption) confers fitness advantages in clonal selection and memory cell persistence. This evolutionary optimization mirrors established biophysical paradigms, such as free energy minimization in protein folding [21], where biological systems adopt thermodynamically favorable states.

While the 3D Wasserstein distance [22] theoretically captures differences in frequency-mutation-size space, computational constraints necessitate projecting distributions onto rank-frequency planes (Python SciPy implementation). This pragmatic reduction decreases the computational cost from $O(n^{3})$ in 3D to $O(n^{2})$ by Sinkhorn approximations and to O(nlogn) after sorting [23]. The results shows that system features are maintained while enabling clinically feasible calculations.

The BCR and TCRβ sequencing profiles were initially processed by representing each sample’s repertoire as a frequency distribution array, where the array dimension corresponds to the number of unique BCR or TCRβ clonotypes and each element contains the occurrence count of specific BCR or TCRβ sequences. A parallel weight array was subsequently generated by squaring the somatic mutation counts of corresponding BCR genes while maintaining identical index positions, with TCR elements uniformly assigned unit weights. Comparative analysis was performed through computational implementation of the Wasserstein metric, where paired distribution-weight arrays from different samples served as input matrices for the distance calculation framework (Table. S3 and Code in Github).

The IgM subtype predominantly operates during initial immune responses before undergoing class switching to IgG/IgA subtypes with specialized effector functions (e.g., mucosal protection by secretory IgA, immune cell recruitment via IgG Fc regions). By modeling these evolutionarily coordinated subtypes as basis vectors in an immune state coordinate system, their biological interdependencies manifest geometrically: The IgM axis exhibits non-zero projections onto IgG/IgA axes due to its irreversible differentiation into these subtypes during antibody maturation, whereas IgG and IgA axes remain mutually orthogonal as they represent terminal differentiation states without interconversion. This mathematically formalizes the biological reality of hierarchical immune maturation pathways, where coordinate system non-orthogonality directly mirrors the unidirectional developmental relationships between antibody subtypes. We compute subtype-specific Wasserstein distances and address their non-orthogonality (from class-switching interdependencies) through Chebyshev norm synthesis [24]. For subtypes associated with autoimmunity like IgE, maximum distance imputation ensures conservative estimates (Eq. 2), which accommodates incomplete clinical profiling while retaining immunological interpretability.

(2) $$D_{\text{total}}=\max\left(d_{\text{IgA}},d_{\text{IgM}},d_{\text{IgG}},d_{\text{TCR}\beta }\right)$$

### 2.2 Experimental methods

#### 2.2.1 Immunization and sample collection

50ug VLP or 2mg CAWS was dissolved in PBS and given to each mouse through intraperitoneal injection. Mice were sacrificed at 3, 7, 14, and 28 days following VLP injection, and at 14 days following CAWS injection. Spleens were collected and homogenized to extract RNA. 3-5ml peripheral blood samples were collected from participants in hospital, and after isolating mononuclear cells, total RNA was extracted.

#### 2.2.2 Experimental workflow and HTS sequencing

Total RNA was extracted from the sample using Trizol according to the user manual. Template Switch PCR with UMIs was performed for reverse transcription [25]. The cDNA products were amplified by nested-PCR with corresponding specific primers. PCR products were purified by DNA magnetic beads. Sequencing was carried out using an Illumina 2x300 pari-end configuration. IgA, IgM, IgG, TCRβ chain data were sequenced independently.

#### 2.2.3 Data preprocessing

Data preprocessing workflow includes QC, trim, alignment, annotation, and UMI deduplicating process. Raw sequences were first checked by FastQC [26], then trimmed by Trimmomatic [27] (Q value ≥ 20). Sequences were annotated to the IMGT reference database [28]. Only the mutations in the V regions are counted for accuracy. Sequences with Levenshtein distances less than 3bp as well as same UMI, V allele, J allele, and CDR3 length were considered as PCR duplicates.

#### 2.2.4 Ethical approval declarations

All mice were kept in SPF environment and fed *ad lib* in Jiangsu Province Academy of Traditional Chinese Medicine. All animal studies have been approved by Ethics Committee of Nanjing University of Chinese Medicine and performed in accordance with the ethical standards (AEWC-20220329-198).

Studies for the colorectal cancer and healthy adults have been approved by Ethics Committee of Nanjing Hospital of Traditional Chinese Medicine (KY2024025).

Studies for the Kawasaki disease patients and healthy children have been approved by Ethics Committee of Soochow University (SUDA20220906A01).

Methods were carried out in accordance with the relevant guidelines and regulations. Informed consent was obtained from all participants.

#### 2.2.5 The inclusion and exclusion criteria

The samples in the study were selected from natural population cohorts, with the criteria below:

1. Initial diagnosis of the corresponding disease for study inclusion;

2. Participants voluntarily join this study and sign an Informed Consent Form (ICF);

3. Exclude participants with a history of cancer;

4. Exclude participants whose immune function is affected by long-term medication use;

5. Exclude participants diagnosed with more than one primary disease simultaneously.

6. Exclude participants with pregnant or lactating women;

7. Exclude participants who have previously participated in other clinical trials and have not yet completed the trial.

## 3 Results

### 3.1 Unsupervised clustering of mouse and clinical data

Pairwise repertoire distance matrices were subjected to t-SNE projection for unsupervised visualization (Fig. 2a, Extended Data Fig. 6). Clinical labels were withheld during clustering and subsequently overlaid for validation, revealing self-organized groupings that precisely matched diagnostic categories. Murine models exhibited antigen-specific clustering patterns: CAWS-induced vasculitis and VLP immunization cohorts formed distinct topological domains (Fig. 2b), while longitudinal tracking of VLP responses demonstrated progressive repertoire evolution across days 3–28 (Fig. 2c). This temporal resolution enabled trajectory reconstruction of immune activation cascades, suggesting potential utility in monitoring subclinical progression through repertoire deviation detection.

In human cohorts, colorectal cancer patients segregated completely from healthy controls (Fig. 2d, Extended Data Fig. 5), with cluster boundaries reflecting malignancy-associated immune dysregulation. The model resolved diagnostic ambiguities in Kawasaki disease (KD): three clinically diagnosed febrile patients clustered with KD cases rather than febrile controls (Fig. 2e), indicating immunological states indistinguishable from confirmed KD cases, revealing probable prior misdiagnoses through immune signature analysis. Ontogenic immune maturation manifested as discrete child-adult partitioning (Fig. 2f), quantifying age-related activation baselines for subhealth state detection.

Global analysis demonstrated complete separation across five clinical states (healthy, pediatric, febrile, KD, cancer; Fig. 2g), with clustering fidelity validated by silhouette analysis and Davies-Bouldin indices. This generalized discriminative capacity stems from modeling fundamental immune processes—clonal selection energetics and SHM-driven adaptation—rather than disease-specific biomarkers. The framework supports incremental diagnostic refinement: new samples are classified through distance comparison to reference clusters, analogous to phylogenetic placement in metagenomic analysis.

The biological significance of data points near cluster boundaries may stems from three mechanistic interpretations: (1) Their intermediate centroid distances reflect comparable transition probabilities between distinct immune activation states, representing continuous biological transitions rather than discrete classifications; (2) Individual variability in baseline immune competence (age-related thymic involution, environmental antigen exposure gradients) creates inconsistency between canonical disease/healthy signatures; (3) Concurrent immunological challenges generate hybrid activation patterns manifesting as boundary-hugging points. While our mathematical framework quantifies immune state transition dynamics through multidimensional energy landscapes, it deliberately refrains from clinical diagnostic categorization, akin to establishing microscopy as an observational tool rather than developing an automated microscopy system with integrated diagnostic classification capabilities. The ontological ambiguity of boundary-proximal points therefore necessitates subsequent diagnostic model development leveraging this quantitative foundation.

Traditional supervised classifiers exhibit limited generalizability constrained by training set scope, whereas this process-driven approach achieves open-ended quantification of immune state transitions. Evaluation through intrinsic clustering metrics (vs binary classification rates) aligns with its foundational physics-inspired design—akin to assessing microscope resolution rather than image recognition accuracy.

### 3.2 Subgrouping within the healthy group

The healthy group did not exclude participants with a history of recent infection or vaccination (Post-IV), whose immune status may be influenced by exogenous antigens or medications. The naive healthy group consists of 5 individuals, while the Post-IV group includes 12 individuals. Significant differences in the relationship between mutation and clone frequency were observed (Fig. 1l, Extended Data Fig. 5a), and two distinct clusters could be identified in the unsupervised clustering (Extended Data fig. 5b). However, the information on the infection history of the healthy children group was not collected. The mutation distribution and clustering result within the group exhibits variability (Fig. 1k, Fig. 2e–f, Extended Data fig. 5c). Aftering trimming healthy data, the differences between healthy and cancer have become more pronounced (Extended Data fig. 5D). Variations within HC group are less pronounced than those in compromised cohorts, thereby not impacting the results of unsupervised clustering (Fig. 2d-g).

### 3.3 Speculation on the individual’s overall immune status

The model is practically valuable only when its results accurately reflect an individual’s overall immune status. As this method is distribution based, so we expect the clustering result to be independent of sample size, provided that a certain accuracy threshold is met. Multiple sub-datasets with varying size were resampled from single sequencing data and the sample distance between them were calculated (Fig. 3a). We found the sub-datasets and the overall population holds the same distribution and the distance between them diminishes at a power-law rate with the increase of the sample size, a tenfold increase in data volume corresponds to a single-digit enhancement in the precision of estimating large samples from small samples (Fig. 3b-d), indicating that small data could represent the overall repertoire status of an individual. After calculating the average distance between CRC patients and HC group, we found the minimum amount of sample size to tell whether an individual is healthy or not at 10${}^{4}$ level. Clustering results with all samples’ data size reduced to $1\times 10^{4}$ confirmed our computational results (Fig. 3e, Extended Data Fig. 7). Although the error is amplified due to the reduction in sample size, the unsupervised clustering of each health condition still shows obvious grouping. KD group and febrile group could still be distinguished. Naive HC, Post-IV HC and child group still holds unique clusters. These findings confirm the feasibility of the method to differentiate various immune statuses with minimal sample quantities. Data size normalization is not necessary for repertoire analysis in this method. Datasets could compare to each other directly regardless of sample size as they all can represent the overall macroscopic status of the individual. Differences calculated from datasets with varying sample size ranging from 20k to 120k matched with the normalized situation and clustering results still aligned with clinical groupings (Fig. 2b-g). This gives the feasibility to estimate the overall health status of an individual from small amounts of peripheral blood, even in the level of droplets.

## 4 Discussion and conclusion

This study establishes a generative framework for simulating immune repertoire dynamics and quantifying inter-repertoire divergence through energy-encoded transformation metrics. By recasting clonal proliferation and somatic hypermutation as preferential attachment processes within an energy-based framework, we achieve dynamic modeling of IR evolution with clinical-grade resolution.

This study fundamentally differs from data-mining approaches via its first-principles derivation framework, intentionally omitting traditional training/validation set partitioning as the mathematical relationships governing lymphocyte distributions (e.g., scale-free network topology) were established through axiomatic biological constraints prior to data exposure. The clinical dataset solely serves to validate a priori theoretical predictions regarding immune activation pattern separability across health states, functioning as a singular verification set rather than a training source. While external validation across populations remains an acknowledged limitation, the demonstrated coherence between predicted and observed cluster formations (self-organized through immunological state alignment rather than stochastic grouping) confirms methodological validity at this proof-of-concept stage. Small-sample sufficiency arises from the framework’s deterministic nature: limited sample size suffices to confirm biologically-driven clustering patterns, contrasting with data-driven models requiring large cohorts to overcome inherent variance.

Crucially, our process-driven approach transcends conventional biomarker discovery by mathematically defining ”immune normality” through deductive reasoning rather than population statistics. The model’s capacity to detect subclinical immune perturbations—leveraging the cascade amplification effect where minimal antigen exposure induces measurable repertoire shifts—positions it as a transformative tool for early disease interception and personalized immunotherapy design.

Surprisingly, comparative analysis revealed that simplified ideal gas analogies—where memory cells behave as low-mass, high-mobility particles—reproduced key repertoire distribution patterns observed in our scale-free model. This theoretical convergence across mechanical and dynamic frameworks suggests deep universality in immune self-organization principles. However, current approximations omit critical biological processes (class-switch recombination, memory cell quiescence) that may introduce systemic biases in chronic disease modeling. Future iterations that incorporate grand canonical ensemble formulations and dissipative structure theory could bridge these gaps while preserving computational tractability.

Our framework deliberately utilizes cost-effective bulk HTS data rather than single-cell sequencing, ensuring clinical viability across resource settings. By analyzing natural population cohorts without demographic filtering, we preserve real-world immunological heterogeneity—a design choice validated through blinded stratification of diagnostically ambiguous cases (e.g., Kawasaki disease vs. febrile infections). The model’s input-agnostic architecture accepts any BCR/TCRβ dataset containing mutation profiles, circumventing platform-specific normalization artifacts that plague conventional comparative analyses.

Precision diagnostics now enable early detection of oncologic and autoimmune pathologies through longitudinal monitoring of deviations from patient-specific immune baselines. In therapeutic development, quantitative tracking of repertoire trajectories provides robust metrics for evaluating vaccine efficacy and immunotherapy responsiveness. Concurrently, comparative analysis of immune energy landscapes is unveiling fundamental mechanisms underlying immunological aging and cross-species conservation patterns, bridging basic research with clinical translation.

While demonstrating robust accuracy across acute/chronic disease models, the framework’s universal applicability requires validation in pathogen-specific contexts—particularly viral latency and metastatic cancer microenvironments. Low-cost immune repertoire sequencing could enable real-time monitoring, transforming our approach into a clinical decision-support pillar. By reconceptualizing the immune system as an endogenous biosensor network, this work bridges theoretical immunology and bedside medicine, heralding an era of physics-informed immune intelligence.

## Data and Code Availability

Source code is avaliable at https://github.com/CYX-SAMIR/IR-simulation. HTS data are available upon request.

## CRediT authorship contribution statement

**Yexing Chen:** Conceptualization, Data curation, Formal analysis, Investigation, Methodology, Project administration, Resources, Software, Supervision, Validation, Visualization, Writing – original draft, Writing – review & editing. **Haiwen Ni:** Resources. **Yongjie Li:** Investigation, Validation. **Jin Ma:** Investigation, Validation. **Chen Huang:** Investigation, Validation. **Sixian Yang:** Methodology. **Xiangfei Xie:** Methodology, Writing – review & editing. **Haitao Lv:** Resources. **Min Li:** Resources. **Peng Cao:** Funding acquisition, Project administration, Resources, Supervision, Writing – review & editing.

## Declaration of competing interest

The authors declare the following financial interests/personal relationships which may be considered as potential competing interests.

Peng Cao and Yexing Chen declare patents for the repertoire quantification, fluctuation detection and comparison algorithm in the article.

## Appendix

### A1 Materials and experimental procedures

C57BL/6 mice were purchased from GemPharmatech (Nanjing, China). The VLP capsid protein (Escherichia virus Qbeta, a.a.1-133, Uniprot Id P03615) was purchased from Nanjing Liheng Biotechnology Co., Ltd. The DNA purification beads was purchased from Beckman (AMPure XP, 20962200)

The wetwork and sequencing procedures [25] were performed by the GENEWIZ, lnc.

### A2 CAWS preparation

The CAWS were prepared from Candida albicans strain NBRC1385 [29], [30]. Candida albicans were incubated in C-limiting medium at 26 ${}^{\circ }$C for 2 days at 160 rpm. Add equal volume of ethanol and overnight in 4 ${}^{\circ }$C cold storage. Then we collect the culture by centrifugation, dissolve the precipitate in water, stir for 2 h, centrifuge the mixture again, harvest the soluble part and mix it with an equal volume of ethanol, and let it stand overnight. Finally, we centrifuge the mixture to obtain the precipitate and dry it with acetone, and dissolve the CAWS dried product in 0.9% physiological saline and sterilize under high pressure for later use.

### A3 Core parameters governing repertoire dynamics

Six biologically constrained parameters (four for TCR systems) define immune repertoire evolution. How these parameters influences the repertoire are as follows:

1. Initial antigenic stimulation distribution: Modeled as Gaussian distribution *N*(0, $\sigma^{2}$) truncated to positive values, reflecting VDJ recombination’s stochastic nature (Extended Data Fig. 3a).
2. Responding lymphocyte pool size: Determines rank-frequency distribution geometry—small populations ($N_{\text{initial}}<5k$) yield concave patterns, while larger cohorts ($N_{\text{initial}}>200k$)develop scale-free architectures (Extended Data Fig. 3b–c).
3. SHM probability landscape: Effective mutation rate = $0.7\times 10^{-3}$ bp${}^{-1}$ division ${}^{-1}$ and calculated to 0.24 cell${}^{-1}$ division ${}^{-1}$ [17], with hypermutation decay up to $N_{{\text{Mut}}^{\text{Max}}}=50$ [31].
4. Stimulation signal modulation: Gaussian-distributed perturbations *N*(0, $\sigma^{2}$) per SHM event.
5. Proliferation kinetics: Sigmoidal stimulus-response relationship $P=e^{KS}/\left(1+e^{KS}\right)$ with K>1, ensuring preferential expansion of high-affinity clones [32], functions that exhibit trends consistent with the initial segment of the Sigmoidal curve can achieve similar effects. Functional robustness confirmed across polynomial/exponential implementations (Extended Data Fig. 3d).
6. Clonal expansion dynamics: Iterative cycles model sustained immune challenges, with power law emergence, saturating at a plateau (Extended Data Fig. 3e-f).

The probability P(i) for a new cell to join a clone depends on the existing cell number N and proliferation speed f(i) of the clone, which is

(A.1) $$P\left(i\right)=\frac{N*f(i)}{\sum N*f(i)}$$

where ∑N*f(i) is the partition function.

## Appendix B Supplementary materials

Supplementary Data S1 Supplementary Raw Research Data. This is open data under the CC BY license http://creativecommons.org/licenses/by/4.0/ Supplementary Data S1
