# Supplementary material for: Profiling adaptive immunity: A quantitative framework for immune repertoire dynamics and clinical diagnostics
Source: Fundam Res. 2025 Jun 3;6(3):1966–74. doi: 10.1016/j.fmre.2025.05.011 (PMC13247450; doi:10.1016/j.fmre.2025.05.011)
Supplement: Supplementary Data S1 — Supplementary Raw Research Data. This is open data under the CC BY license http://creativecommons.org/licenses/by/4.0/ [file mmc1.pdf]

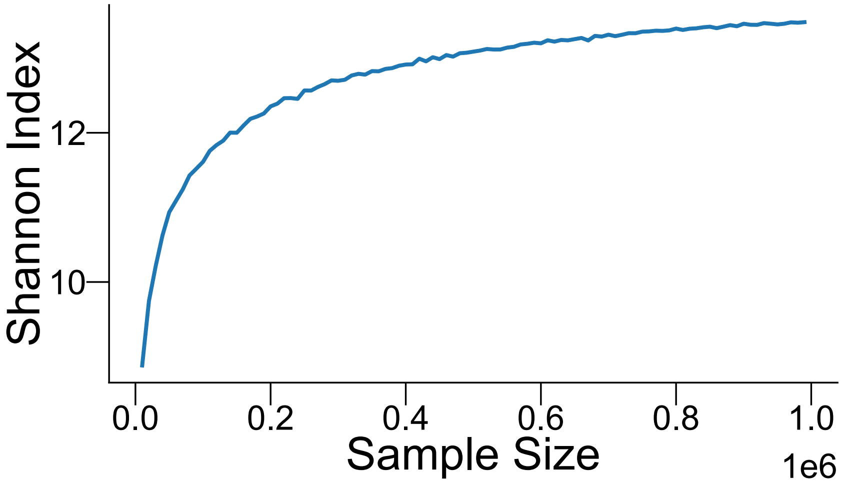

Extended Data Fig. 1: **Correlation between Shannon Index and sample size.** The Shannon Index value increases with the augmentation of the sample size.

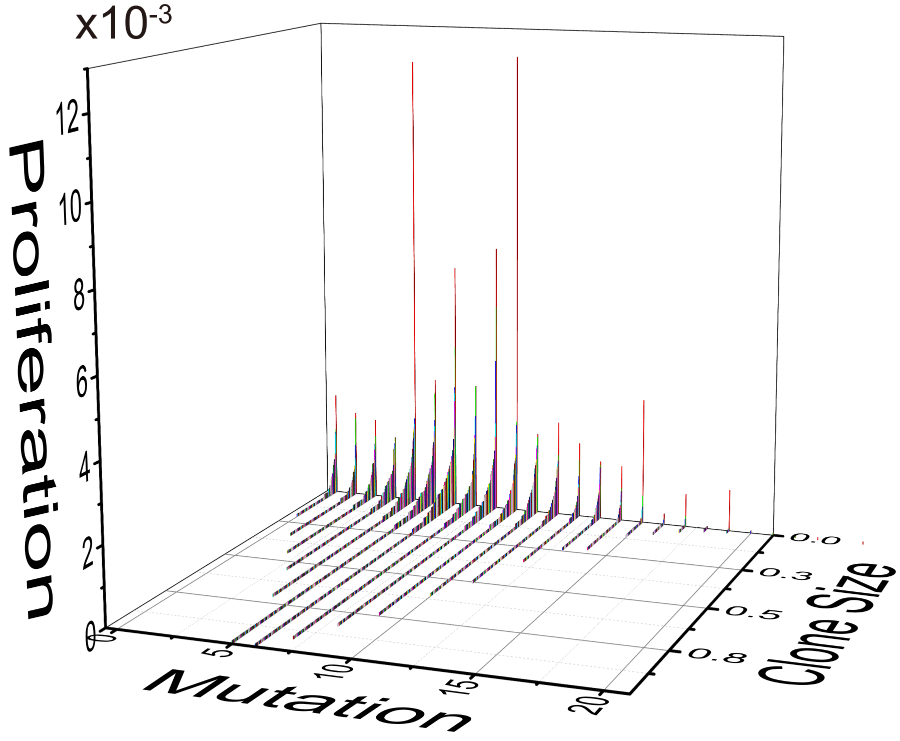

Extended Data Fig. 2: **A 3D structural demonstration of repertoire.** The three basis of the vector space are mutation, proliferation and clone number, each cell in repertoire occupies a point in the space based on its BCR or TCR sequence. The 3D map is uniquely determined by a repertoire.

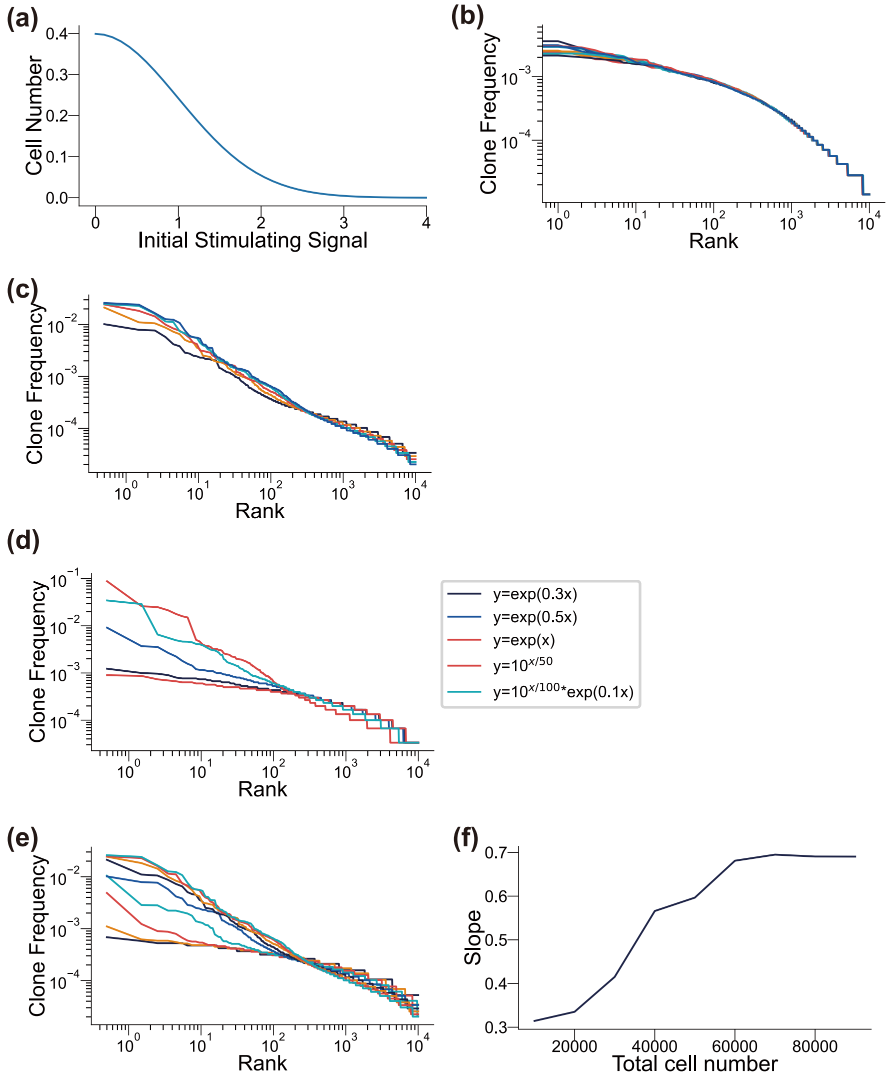

Extended Data Fig. 3: **Parameters affecting the scale-free distribution of simulated repertoire.** (**a**) The number of naive B or T cells with varying binding capacity and generate stimulating signal of different strength to a random antigen should follow a Gaussian distribution. (**b, c**) Simulation starts with 1k-5k cells and proliferate to 200k cells will generate a concave distribution, while power-law distribution emerges when starting with 50-90k cells. (**d**) Different formulations calculating the proliferation speed from stimulating signal can all produce a power law distribution, the greater the derivative of the function, the steeper the corresponding curve is. (**e, f**) The scale-free distribution generated from different final cell numbers range from 10k-100k. As the number of cells increases, the slope of the curve also increases until it reaches a plateau.

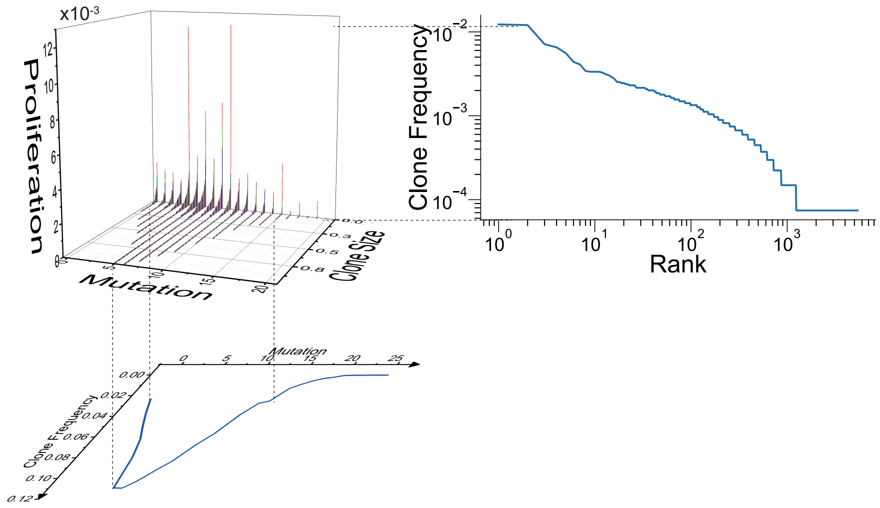

Extended Data Fig. 4: **Correlation between 3D structures and various distributions.** The dimensionality reduction results of the 3D structure at different angles exhibit the unimodal distribution and the scale-free distribution.

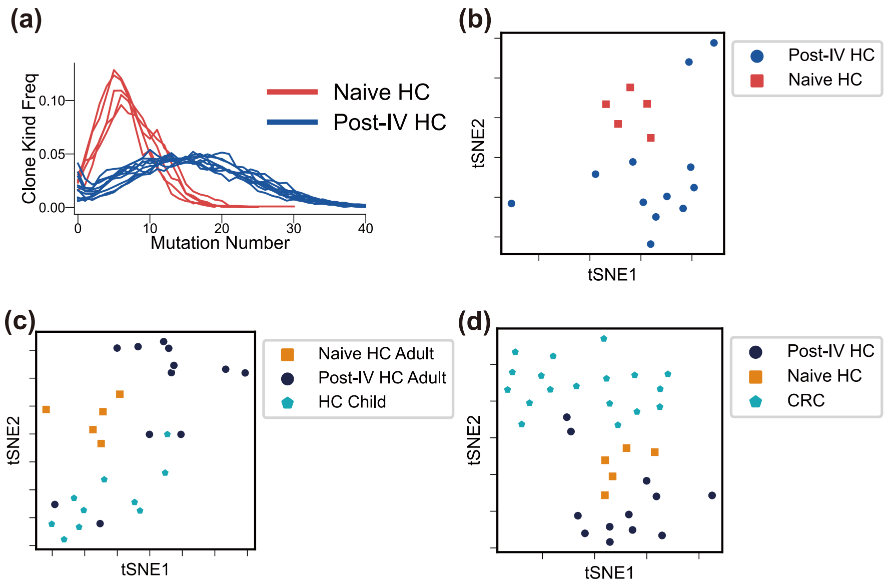

Extended Data Fig. 5: **Subgrouping within the healthy group.** (**a**) The differences in mutation accumulation between naive healthy and Post-IV groups. (**b, c**) The unsupervised clustering of naive healthy, Post-IV group and healthy children group. Post-IV group exhibits fluctuations in clustering due to its diverse immune statuses. (**d**) In comparison to cancer patients, 14 out of 17 healthy individuals exhibited consistent immune statuses, while the remaining 3 displayed immune conditions that were on the boundary between the two states.

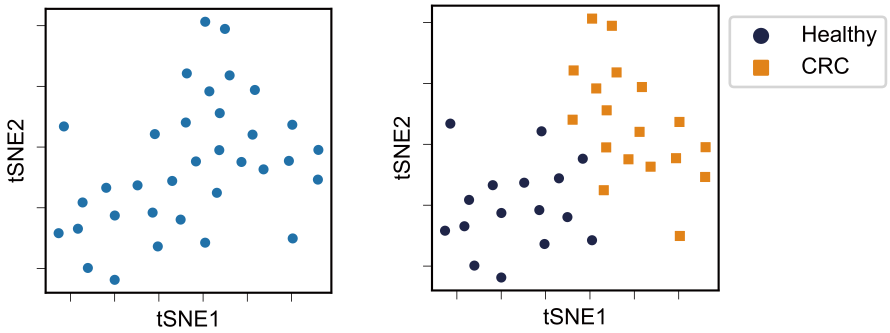

Extended Data Fig. 6: **Blind clustering of repertoire data.** The distances between any two samples were calculated and visualized to produce clustering results. The clinical information of HC and CRC are treated as a blind factor and are subsequently incorporated to verify the accuracy of the cluster.

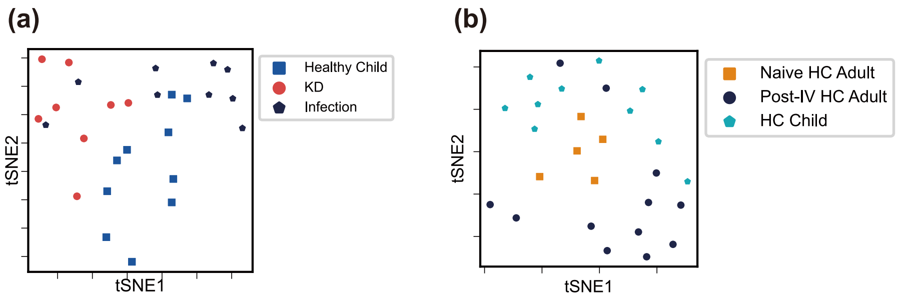

Extended Data Fig. 7: **detailed clustering result of clinical data with sample size reduced to $1{*}10^4$.** (**a**) Distinct difference were observed between KD and febrile group with minimal sample size, the healthy child group demonstrates variations attributed to the persistent immune responses. (**b** difference among naive HC, Post-IV HC and HC child could also be observed with minimal sample size.)

table S1. Clinical information for participants included in the study.

Clinical Diagnosis	Number(M/F)	Age(median)	Sample Type
Colorectal cancer	6/12	30-85(68)	Peripheral blood
Kawasaki disease	5/3	1-10(2)	Peripheral blood
Febrile	7/3	1-9(2)	Peripheral blood
Healthy children	1/9	2-4(3)	Peripheral blood
Healthy adults	5/12	23-32(25)	Peripheral blood

table S2. Immunization information for C57BL/6 mice used in the study.

Antigen	Number	days after immunization	Sample Type
Healthy control	6	/	spleen
CAWS	10	14	spleen
VLP	6	3	spleen
VLP	8	7	spleen
VLP	8	14	spleen
VLP	6	28	spleen

table S3. Python code snippet for repertoire distance calculation with wasserstein distance.

```
from SciPy.stats import wasserstein_distance

example_repertoire_distribution_1 =
    [100,80,60,50,50,30,30,30,10,10,10,10,1,1,1,1,1]
example_repertoire_distribution_2 =
    [130,90,50,50,30,30,30,20,20,5,5,3,3,1,1,1,1]

example_repertoire_mutation_1 =
    [3,5,2,6,8,4,2,4,3,2,1,0,0,0,1,0,0]
example_repertoire_mutation_2 =
    [3,5,2,6,8,4,2,4,3,2,1,0,0,0,1,0,0]

normalized_distribution_1 = [i/sum(
    example_repertoire_distribution_1) for i in
    example_repertoire_distribution_1]
normalized_distribution_2 = [i/sum(
    example_repertoire_distribution_2) for i in
    example_repertoire_distribution_2]

example_repertoire_weight_1 = [i*i+0.1 for i in
    example_repertoire_mutation_1]
example_repertoire_weight_2 = [i*i+0.1 for i in
    example_repertoire_mutation_2]

repertoire_distance = wasserstein_distance(
  normalized_distribution_1,
  normalized_distribution_2,
  example_repertoire_weight_1,
  example_repertoire_weight_2,
)
```
